# Targeting the Mitochondrial Respiratory Chain of *Cryptococcus* through Antifungal Chemosensitization: A Model for Control of Non-Fermentative Pathogens

**DOI:** 10.3390/molecules18088873

**Published:** 2013-07-25

**Authors:** Jong H. Kim, Ronald P. Haff, Natália C. G. Faria, Maria de L. Martins, Kathleen L. Chan, Bruce C. Campbell

**Affiliations:** 1 Plant Mycotoxin Research Unit, Western Regional Research Center, USDA-ARS, 800 Buchanan St., Albany, CA 94710, USA; E-Mails: ron.haff@ars.usda.gov (R.P.H.); kathy.chan@ars.usda.gov (K.L.C.); campbellbrc@gmail.com (B.C.C.); 2 Instituto de Higiene e Medicina Tropical/CREM, Universidade Nova de Lisboa, Portugal; E-Mails: natalia.faria@arslvt.min-saude.pt (N.C.G.F.); luzmartins@ihmt.unl.pt (M.L.M.)

**Keywords:** chemosensitization, *Cryptococcus*, *Candida*, *Saccharomyces*, octyl gallate, 2,3-dihydroxybenzaldehyde, mitochondrial respiration inhibitors

## Abstract

Enhanced control of species of *Cryptococcus*, non-fermentative yeast pathogens, was achieved by chemosensitization through co-application of certain compounds with a conventional antimicrobial drug. The species of *Cryptococcus* tested showed higher sensitivity to mitochondrial respiratory chain (MRC) inhibition compared to species of *Candida*. This higher sensitivity results from the inability of *Cryptococcus* to generate cellular energy through fermentation. To heighten disruption of cellular MRC, octyl gallate (OG) or 2,3-dihydroxybenzaldehyde (2,3-DHBA), phenolic compounds inhibiting mitochondrial functions, were selected as chemosensitizers to pyraclostrobin (PCS; an inhibitor of complex III of MRC). The cryptococci were more susceptible to the chemosensitization (*i.e.*, PCS + OG or 2,3-DHBA) than the *Candida* with all *Cryptococcus* strains tested being sensitive to this chemosensitization. Alternatively, only few of the *Candida* strains showed sensitivity. OG possessed higher chemosensitizing potency than 2,3-DHBA, where the concentration of OG required with the drug to achieve chemosensitizing synergism was much lower than that required of 2,3-DHBA. Bioassays with gene deletion mutants of the model yeast *Saccharomyces cerevisiae* showed that OG or 2,3-DHBA affect different cellular targets. These assays revealed mitochondrial superoxide dismutase or glutathione homeostasis plays a relatively greater role in fungal tolerance to 2,3-DHBA or OG, respectively. These findings show that application of chemosensitizing compounds that augment MRC debilitation is a promising strategy to antifungal control against yeast pathogens.

## 1. Introduction

Mycotic infectious diseases, such as candidiasis or cryptococcosis caused by various species of *Candida* or *Cryptococcus*, respectively, are continuously expanding as serious global health issues. This expansion is mainly associated with immunocompromised disorders (e.g., AIDS, chemotherapy, radiotherapy, *etc*.) [1] and concomitant development of resistance to antifungal drugs [2]. Consequently, there is persistent need to enhance the effectiveness of conventional antimycotic drugs or discover and develop new ones [3].

**Figure 1:**
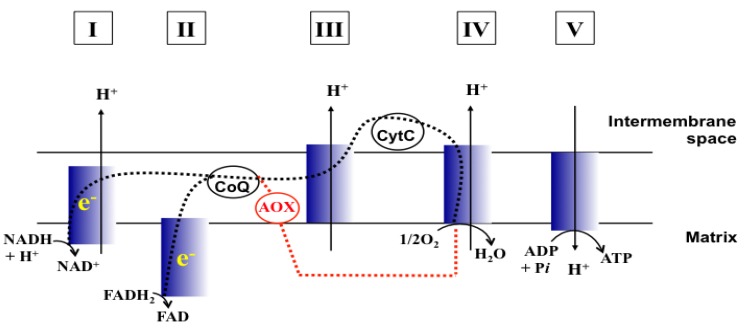
Schematic representation of MRC (Adapted from [17] and [18]). CoQ, Coenzyme Q; CytC, Cytochrome C; e^−^, Electrons; Dashed line (black), Electron flow w/o MRC inhibition; Dashed line (red), Electron flow through AOX when MRC is inhibited. I to V, complexes I to V of MRC.

Mitochondrial functions of fungi have been examined as potential targets for antifungal therapy ([4] for review). For example, certain mitochondrial mutants exhibited increased susceptibility to polyene or azole drugs, possibly resulting from changes in sterol levels of membranes ([4] and references therein). In particular, the mitochondrial respiratory chain (MRC; See Figure 1 for the structure of MRC) is a target of the MRC-inhibitory drug atovaquone (ATQ; hydroxy-1,4-naphthoquinone) for control of the infections of fungi, such as *Pneumocystis jirovecii* (pneumonia) [5]. Such MRC-inhibitory drugs disrupt production of cellular energy (ATP) in fungal cells. Of note, ATQ is also used to treat malarial parasites, such as *Plasmodium*, where ATQ not only inhibits the MRC, but also disrupts the inner mitochondrial membrane potential (ΔΨ_m_) [6].

MRC inhibitors can also trigger oxidative stress resulting from leakage of electrons from the MRC, resulting in oxidative damage to cellular components, such as cell membranes/lipid bilayers. This demonstrates that the fungal antioxidant system (superoxide dismutases, glutathione reductase, stress signaling pathway, *etc*.) plays a crucial role in maintaining cellular integrity from toxic reactive oxygen species [7,8]. To date, while the MRC has been targeted for control of agro-fungal pathogens, it has been a relatively unexploited drug target against clinical fungal pathogens.

Antifungal MRC targeting could possibly be enhanced by compounds affecting cellular redox homeostasis. Natural phenolic compounds or their structural derivatives, which are redox-active, can serve as potent redox cyclers against fungal pathogens, resulting in inhibition of microbial growth ([9] and references therein). This inhibition results from destabilization of cellular redox homeostasis, antioxidant systems or the function of redox-sensitive components [10,11]. Meanwhile, certain phenolic compounds/derivatives can also inhibit various functions of mitochondrial components (See Table 1). For example, gallate derivatives, such as propyl- or octyl gallate (PG or OG, respectively), inhibit the activity of cellular alternative oxidase (AOX). AOX functions in fungi to overcome the toxicity triggered by MRC inhibitors, rendering the completion of electron transfer *via* MRC ([12,13]; See Figure 1). OG further disrupts and/or disorganizes the lipid bilayer-protein interface in fungal cells [14]. Likewise, acetylsalicylic acid (AcSA) or 2,3-dihydroxybenzaldehyde (2,3-DHBA) also inhibits the functions of mitochondria or mitochondrial superoxide dismutase (Mn-SOD), respectively, in fungi [15,16].

Chemosensitization is a strategy where co-application of certain types of compounds along with a conventional antimicrobial drug increases the effectiveness of the drug [19,20,21,22]. Examples include: (1) 4-methoxy-2,3,6-trimethylbenzensulfonyl-substituted d-octapeptide, which sensitizes pathogenic *Candida* strains to fluconazole (FLC), resulting in countering FLC resistance of clinical isolates [22], (2) 7-chlorotetrazolo[5,1-c]benzo[1,2,4]triazine (CTBT), which increases the susceptibility of *Candida* and *Saccharomyces* strains to cycloheximide, 5-fluorocytosine and azole drugs [23], (3) squalamine (a modifier of membrane integrity by increasing permeability of drugs), which enhances the susceptibility of various antibiotic-resistant and susceptible strains of Gram-negative bacteria to drugs [21], and (4) antimycin A (AntA) and benzhydroxamic acid (BHAM), which makes *Rhizopus oryzae* hypersensitive to triazoles, *i.e.*, posaconazole (PCZ) and itraconazole (ICZ), *via* apoptosis [24]. Collectively, these studies showed that antimicrobial drug therapy that includes chemosensitization could lead to lowering dosage levels of conventional drugs needed for control of pathogens, in both drug-resistant and susceptible strains.

Various species of *Cryptococcus* and *Candida* are human and animal pathogens. For example, cryptococcal meningitis is reported to be the leading cause of death among those infected with HIV [25]. However, one of the key differences between the yeasts in these two genera is that species of *Cryptococcus* are non-fermentative, while the *Candida* species are fermentative [16].

**Table 1:** Mitochondrial targets and structures of compounds tested in this study.

Compounds	Target	References
**2,3-DHBA**	Mn-SOD ( *Saccharomyces cerevisiae* Mn-SOD gene deletion mutant is hypersensitive to 2,3-DHBA)	[ 15]
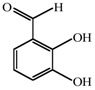		
**AcSA**	General mitochondrial function ( *Aspergillus fumigatus sakA*Δ [oxidative stress-responsive mitogen-activated protein kinase gene deletion mutant] was also hypersensitive to AcSA [See [26]].)	[ 16]
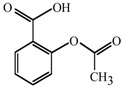		
**PG**	AOX	[ 13,27]
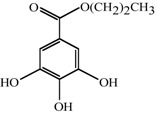		
**OG**	AOX	[ 28,29]
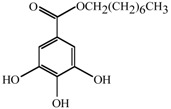		

Based on this difference, we reasoned the following: (1) When cellular MRC is disrupted by MRC-inhibitory drug(s), the *Cryptococcus* would show higher sensitivity than the *Candida*, (2) This higher sensitivity is due to the fact that the *Candida* can generate cellular energy also through fermentation (other than MRC), while the *Cryptococcus*, being non-fermentative, lack this ability, and (3) Thus, MRC could serve as an effective antifungal target especially for control of *Cryptococcus* pathogens.

In this study, we investigated if selected phenolic compounds/derivatives (See Table 1) could enhance the antifungal potency of pyraclostrobin (PCS), the most potent complex III inhibitor of MRC in our test, against *Cryptococcus*. Our hypothesis was that co-application of phenolic compounds/derivatives (as chemosensitizers) and PCS will negatively affect the common cellular target, *i.e.*, functions of mitochondria, resulting in increased sensitivity of fungi. We also evaluated the potential of these chemosensitizing compounds to serve as active pharmaceutical “leads” against *Cryptococcus* yeasts, and compared the effectiveness of chemosensitization between *Cryptococcus* and *Candida* (See Table 2 for strains tested). Our results showed that the *Cryptococcus* were more susceptible to OG- or 2,3-DHBA-mediated chemosensitization to PCS than the *Candida*, where the chemosensitizing capacity of OG was found to be greater than that of 2,3-DHBA.

**Table 2:** Yeast strains used in this study.

Yeast strains	Strain characteristics	Source/Reference
*Cryptococcus*		
*C. neoformans *90112	Clinical reference strain	ATCC ^a^
*C. neoformans *208821	Clinical isolate	ATCC
*C. neoformans *MYA-4564	Clinical reference strain	ATCC
*C. neoformans *MYA-4565	Clinical reference strain	ATCC
*C. neoformans *MYA-4566	Clinical reference strain	ATCC
*C. neoformans *MYA-4567	Clinical reference strain	ATCC
*C. neoformans *CN24	Clinical isolate	IHMT ^b^
*C. gatti *MYA-4560	Clinical reference strain	ATCC
*C. gatti *MYA-4561	Clinical reference strain	ATCC
*Candida*		
*C. albicans *90028	Clinical reference strain	ATCC
*C. albicans *CAN242	Clinical isolate	IHMT
*C. albicans *CAN276	Clinical isolate	IHMT
*C. glabrata *90030	Clinical reference strain	ATCC
*C. glabrata *2001	Clinical reference strain	ATCC
*C. glabrata *CAN252	Clinical isolate	IHMT
*C. krusei *6258	Clinical reference strain	ATCC
*C. krusei *CAN82	Clinical isolate	IHMT
*C. krusei *CAN75	Clinical isolate	IHMT
*Saccharomyces*		
*S. cerevisiae *BY4741	Model yeast, Parental strain (Mat a *his3*∆*1 leu2*∆*0 met15*∆*0 ura3*∆*0*)	SGD ^c^
*S. cerevisiae sod2*∆	Mitochondrial superoxide dismutase (Mn-SOD) mutant derived from BY4741	SGD
*S. cerevisiae yap1*∆	Transcription factor *YAP1* mutant derived from BY4741	SGD
*S. cerevisiae trr1*∆	Cytosolic thioredoxin reductase mutant derived from BY4741	SGD
*S. cerevisiae trr2*∆*S. cerevisiae tsa1*∆	Mitochondrial thioredoxin reductase mutant derived from BY4741Thiredoxin peroxidase mutant derived from BY4741	SGD SGD

^a.^ATCC, American Type Culture Collection, Manassas, VA, USA; ^b.^IHMT, Instituto de Higiene e Medicina Tropical/CREM, Universidade Nova de Lisboa, Portugal; ^c.^SGD, *Saccharomyces* Genome Database [30].

## 2. Results and Discussion

We initially tested the effect of chemosensitization by co-applying commercial antifungal/antimalarial drugs “ATQ + proguanil” on the growth of fermenting and non-fermenting yeast pathogens. For this test, we chose representative yeast pathogens, *i.e.*, *C. albicans* 90028 as a fermentor, and *C. neoformans* 90112 and *C. gatti* 4560 as non-fermentors. In protozoan parasites, co-application of proguanil (a mitochondria-modulating chemosensitizer) increased anti-parasitic activity of ATQ [31]. Noteworthy is that proguanil-mediated chemosensitization was specific for ATQ. Proguanil did not increase the potency of other types of MRC inhibitors (e.g., myxothiazole, AntA) [31]. Thus, these results with malarial parasites (*Plasmodium*) indicated “drug-chemosensitizer specificity” existed during the chemosensitization process.

We used the checkerboard microdilution bioassay protocol outlined by the European Committee on Antimicrobial Susceptibility Testing (EUCAST) [32], with various concentrations of ATQ (0.25, 0.5, 1, 2, 4, 8, 16 μg/mL) and proguanil (0.25, 0.5, 1, 2, 4, 8, 16 μg/mL). Our results showed that: (1) Independent application of ATQ or proguanil, alone, did not exhibit discernable growth inhibition in any of the test yeast strains, even at the highest concentration (*i.e.*, 16 μg/mL), and also (2) Co-application of ATQ with proguanil did not enhance the antifungal activity of either compounds, indicating no chemosensitization occurred by “ATQ + proguanil” co-treatment in these strains (Data not shown).

In the model yeast *Saccharomyces cerevisiae*, nine cellular transporters required for sequestering toxic drugs/compounds out of the cell need to be knocked out to exhibit ATQ sensitivity [33]. This indicates active drug-detoxification systems do operate in *S. cerevisiae*. We surmised that pathogenic yeasts, *i.e.*, *Cryptococcus* or *Candida*, might also operate similar type(s) of detoxification system(s), enabling these pathogens to escape from ATQ/proguanil-triggered toxicity. Therefore, we performed chemosensitization tests using other types of MRC inhibitors with co-application of phenolic compounds (*i.e.*, OG, PG, 2,3-DHBA, AcSA; See Table 1) as chemosensitizers.

### 2.1. PCS Is the Most Potent MRC Inhibitor in *Cryptococcus*

First, we identified the potency of MRC inhibitors tested against yeast pathogens. Antifungal efficacy was compared among 12 different MRC inhibitors disrupting one of the five different components of MRC, *i.e.*, complexes I to IV or AOX (See Table 3, Figure 1). The level of differential sensitivity between fermenting (*Candida*) and non-fermenting (*Cryptococcus*) yeasts to the MRC inhibitors was determined by an agar plate-based yeast dilution bioassay (See Experimental section). We initially examined (1) three fermentors: *C. albicans* 90028, *C. glabrata* 90030, *C. krusei* 6258 and (2) two non-fermentors: *C. neoformans* 90112, *C. gatti* MYA-4560.

**Figure 2:**
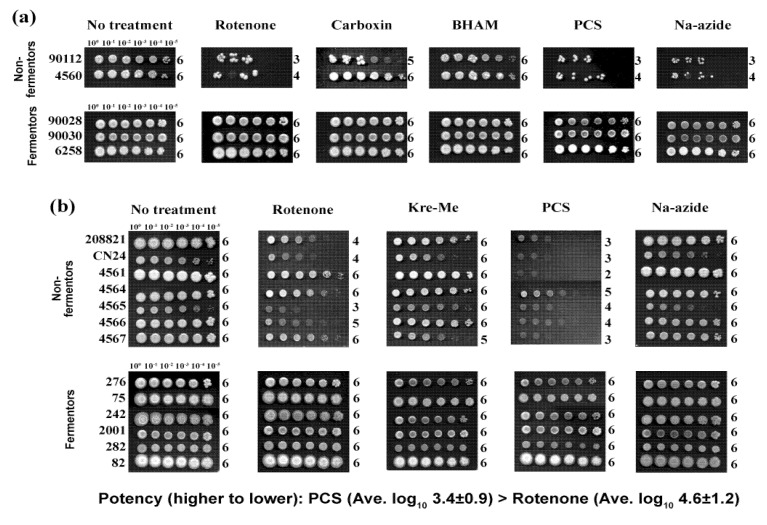
Differential antifungal activity of MRC inhibitors, targeting complexes I - IV or AOX, in yeast pathogens. (**a**) Representative yeast dilution bioassay showing *Cryptococcus* strains, non-fermentors, are relatively more sensitive to MRC inhibitors (rotenone, carboxin, PCS, Na-azide) than *Candida* strains, fermentors. 10^0^ to 10^−5^, yeast-cell dilution level; Numbers on the right side of each row (0 to 6), log_10_ score of cell numbers visible (survived) (See Experimental section and Table 3). (**b**) Yeast dilution bioassay showing PCS is the most potent MRC inhibitor tested, followed by rotenone (Cell dilution level showing visible growth equates to antifungal potency [higher dilution score with visible growth = lower potency]; PCS, average log_10_ score = 3.4 ± 0.9 *vs.* rotenone, average log_10_ score = 4.6 ± 1.2. Average log_10_ score was determined in nine *Cryptococcus* strains). As observed in panel (a), *Candida* strains did not exhibit growth inhibition to any of the MRC inhibitors tested.

MRC inhibitors targeting complex I, III or IV reduced the growth of the *Cryptococcus*, with differing levels of fungal sensitivity (Table 3, Figure 2a). For example, when *C. neoformans* 90112 was treated with complex III inhibitors, growth was inhibited by 100 to 1000 times more compared to controls (*i.e.*, log_10_ dilution score of “no treatment” controls was “6” [*i.e.*, yeast cells appeared at the highest dilution level of 10^6^] *vs*. log_10_ score of “treatments” was “3–4” [*i.e.*, cells did not appear at dilution levels greater than10^3^–10^4^]), depending on types of complex III inhibitors applied. Also, rotenone (a complex I inhibitor) and Na-azide (a complex IV inhibitor) inhibited the growth of *C. neoformans* 90112 at cell dilution levels above 10^3^ times.

Growth of *C. gatti* 4560 was also decreased by four different MRC inhibitors (Table 3). However, unlike the results of *C. neoformans* 90112, AntA and AZS did not inhibit the growth of *C. gatti* 4560 (Table 3). Moreover, besides carboxin, which inhibited *C. neoformans* 90112, both complex II and AOX inhibitors did not discernably inhibit the growth of *C. neoformans* 90112 or *C. gatti* 4560 (See also Figure 2a).

**Figure 3:**
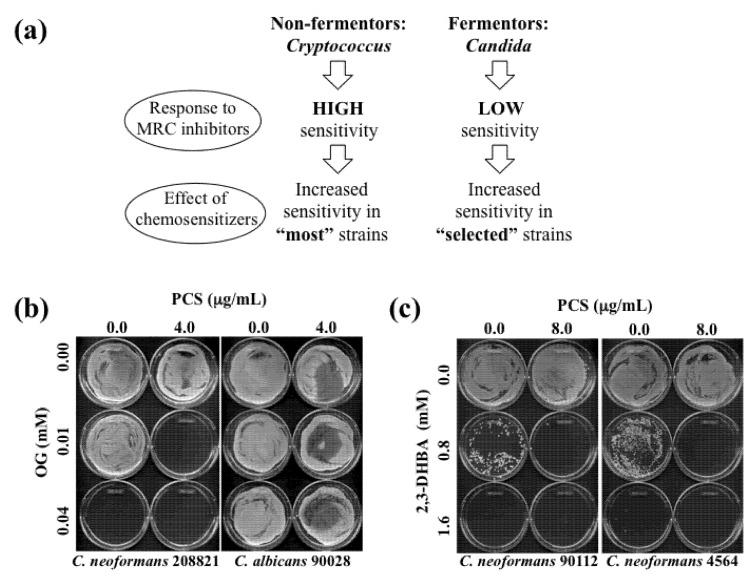
(**a**) Diagram describing effects of OG- or 2,3-DHBA-mediated chemosensitization to PCS in “most” *Cryptococcus* strains (non-fermentors) or in “selected (e.g., five sensitive strains described in Table 6, Table 8)” *Candida* strains (fermentors). (**b**) Representative MFC test results performed in *C. neoformans* 208821 and *C. albicans* 90028 after chemosensitization (PCS + OG). Results showed that *C. neoformans* 208821 (non-fermentor) was more sensitive (*i.e.*, “no growth” with PCS_4.0 μg/mL_ + OG_0.01 mM_) to the chemosensitization than *C. albicans* 90028 (fermentor) (*i.e.*, “growth” with PCS_4.0 μg/mL_ + OG_0.01 mM_). (**c**) Representative MFC test results performed in *C. neoformans* 90112 and 4564 after chemosensitization (PCS + 2,3-DHBA).

Based on these initial bioassays, rotenone, Kre-Me, PCS and Na-azide (exhibiting antifungal activity against both *C. neoformans* 90112 and *C. gatti* 4560) were selected for further evaluation for antifungal potency against additional test strains (*i.e.*, seven additional *Cryptococcus* and six additional *Candida* strains) (See Figure 2b). A similar trend of growth inhibition was found in these additional *Cryptococcus* strains with PCS and rotenone, while Kre-Me and Na-azide showed almost no effect. The growth of the additional *Candida* strains was not noticeably affected by any of these same treatments. In summary: (1) PCS possessed the highest antifungal activity (Average log_10_ dilution score = 3.4 ± 0.9, this lowest average log_10_ score was based upon the results using nine *Cryptococcus* strains shown in Figure 2a,b), followed by rotenone (average log_10_ score = 4.6 ± 1.2); (2) as expected, the growth of the *Candida* (fermenting yeasts) was not affected by any of the MRC inhibitors tested (Table 3; Figure 2a,b), and (3) thus, we chose PCS as the most potent MRC-inhibitory drug against *Cryptococcus* in our chemosensitization study.

**Table 3:** Summary of levels of growth inhibition of representative yeast pathogens by MRC inhibitors: agar plate-based yeast dilution bioassays. ^a^

MRC components targeted	MRC inhibitors applied	*C. neoformans* 90112	*C. gatti* MYA-4560	*C. albicans* 90028	*C. glabrata* 90030	*C. krusei* 6258
	None	6	6	6	6	6
I	Rotenone	**3**	**4**	6	6	6
II	Carboxin	**5**	6	6	6	6
	TTFA	6	6	6	6	6
	3-NPA	6	6	6	6	6
AOX	BHAM	6	6	6	6	6
	SHAM	6	6	6	6	6
III	AntA	**4**	6	6	6	6
	Kre-Me	**3**	**5**	6	6	6
	PCS	**3**	**4**	6	6	6
	AZS	**4**	6	6	6	6
IV	KCN	6	6	6	6	6
	Na-azide	**3**	**4**	6	6	6

^a.^TTFA, Thenoyltrifluoroacetone; 3-NPA, 3-Nitropropionic acid; BHAM, Benzhydroxamic acid; SHAM, Salicylhydroxamic acid; AntA, Antimycin A; Kre-Me, Kresoxim methyl; PCS, Pyraclostrobin; AZS, Azoxystrobin; KCN, Potassium cyanide; Na-azide, Sodium azide. Numbers represented highest dilution level (log_10_) where cell growth was visible. Numbers in bold show cell growth was inhibited (*viz*., < 6).

### 2.2. Antifungal Chemosensitization Tests in Yeast Pathogens

#### 2.2.1. Selection of OG and 2,3-DHBA as the Most Potent Chemosensitizers

Next, we identified the most potent antifungal chemosensitizer(s), among four phenolic compounds listed in Table 1, which inhibit functions of fungal mitochondria. Based on agar plate-based yeast dilution bioassay (See Experimental section), we determined minimum inhibitory concentrations (MICs) of OG, PG, 2,3-DHBA and AcSA. As shown in Table 4, OG and 2,3-DHBA possessed the highest antifungal activity (*i.e.*, low MIC values) compared to PG or AcSA. Of note, *Candida* strains were generally more tolerant to the phenolic compounds tested compared to *Cryptococcus* strains. Based on these results, OG and 2,3-DHBA were selected as the chemosensitizers to test with PCS.

**Table 4:** MIC (mM) of four phenolic compounds tested in this study: agar plate-based yeast dilution bioassays ^a^.

Strains	OG	PG	2,3-DHBA	AcSA
*C. neoformans* 90112	0.1	> 10	0.2	7
*C. gatti* 4560	0.1	> 10	0.3	7
*C. albicans* 90028	0.3	> 10	1.0	> 10
*C. glabrata* 90030	0.2	> 10	2.0	> 10
*C. krusei* 6258	0.3	> 10	2.0	> 10
*Average*	0.2 ± 0.1	> 10	1.1 ± 0.9	> 8.8 ± 1.6

^a^ MIC: Minimum inhibitory concentration, where no yeast growth was observed in any dilution spots on the agar plate.

#### 2.2.2. Chemosensitization in *Cryptococcus*: 2,3-DHBA + PCS

Antifungal chemosensitization was tested based on the EUCAST checkerboard microdilution bioassay protocol [32] (concentrations of compound examined were listed in Experimental section). For MICs, “synergistic” Fractional Inhibitory Concentration Indices (FICIs; see Experimental section for calculations) (*i.e.*, FICI ≤ 0.5) were found between 2,3-DHBA and PCS for most *Cryptococcus* strains (Table 5). Despite the absence of calculated synergism, as determined by “indifferent” interactions [34] (Table 5), there was increased antifungal activity of 2,3-DHBA and PCS (*i.e.*, chemosensitization; FICI = 0.8) in *C. neoformans* 4567*,* which was reflected in lowered MICs of 2,3-DHBA or PCS when combined. However, for Minimum Fungicidal Concentrations (MFCs), “synergistic” Fractional Fungicidal Concentration Indices (FFCIs) (at the level of ≥ 99.9% fungal death) between 2,3-DHBA and PCS occurred only in *C. neoformans* strains 4565 and CN24 (Table 5). Therefore, results indicated that the 2,3-DHBA-mediated chemosensitization with PCS is fungistatic, not fungicidal (*i.e.*, Mean MFC_combined_ for 2,3-DHBA or PCS/ Mean MIC_combined_ for 2,3-DHBA or PCS was > 4 [See [35] for reference]), in most *Cryptococcus* strains tested.

**Table 5:** Antifungal chemosensitization of 2,3-DHBA (mM) to PCS (μg/mL) tested against *Cryptococcus* strains: EUCAST-based microdilution bioassays. ^a^

Strains	Compounds	MIC	MIC	FICI
		alone	combined	
*C. neoformans* 90112	2,3-DHBA	0.2	0.05	**0.5**
	PCS	4	1	
*C. neoformans* 208821	2,3-DHBA	0.2	0.05	**0.3**
	PCS	>16 ^b^	2	
*C. neoformans* 4564	2,3-DHBA	0.2	0.05	**0.5**
	PCS	>16	8	
*C. neoformans* 4565	2,3-DHBA	0.1	0.05	**0.5**
	PCS	>16	1	
*C. neoformans* 4566	2,3-DHBA	0.2	0.05	**0.3**
	PCS	>16	2	
*C. neoformans* 4567	2,3-DHBA	0.2	0.05	0.8
	PCS	4	2	
*C. neoformans* CN24	2,3-DHBA	0.1	0.05	**0.5**
	PCS	16	0.25	
*C. gatti* 4560	2,3-DHBA	0.1	0.05	**0.5**
	PCS	8	0.25	
*C. gatti* 4561	2,3-DHBA	0.2	0.05	**0.5**
	PCS	4	1	
Mean	2,3-DHBAPCS	0.218.2	0.051.9	**0.4**
*t*-test ^c^	2,3-DHBA	-	*p *< 0.005	-
	PCS	-	*p *< 0.005	-
*C. neoformans *90112	2,3-DHBA	1.6	0.8	0.6
	PCS	>16	2	
*C. neoformans *208821	2,3-DHBA	0.8	0.8	2.0
	PCS	>16	>16	
*C. neoformans *4564	2,3-DHBA	1.6	0.8	0.6
	PCS	>16	4	
*C. neoformans *4565	2,3-DHBA	1.6	0.4	**0.5**
	PCS	>16	8	
*C. neoformans *4566	2,3-DHBA	1.6	0.8	0.8
	PCS	>16	8	
*C. neoformans *4567	2,3-DHBA	0.8	0.8	2.0
	PCS	>16	>16	
*C. neoformans* CN24	2,3-DHBA	3.2	1.6	**0.5**
	PCS	>16	0.25	
*C. gatti* 4560	2,3-DHBA	0.4	0.2	1.0
	PCS	>16	16	
*C. gatti* 4561	2,3-DHBA	1.6	0.8	0.6
	PCS	>16	2	
Mean	2,3-DHBA	1.5	0.8	0.9
	PCS	32.0	11.6	
*t*-test	2,3-DHBA	-	*p *< 0.05	-
	PCS	-	*p *< 0.005	-

^a^ Synergistic FICIs and FFCIs are indicated in bold; ^b^ PCS was tested up to 16 μg/mL. For calculation purposes, 32 μg/mL (doubling of 16 μg/mL) was used; ^c^ Student’s *t*-test for paired data, mean MIC or MFC of each compound (combined, *i.e*., chemosensitization) *vs*. mean MIC or MFC of each compound (alone, *i.e*., no chemosensitization), was determined in nine strains (Calculation was based on [36]).

#### 2.2.3. Chemosensitization in *Candida*: 2,3-DHBA + PCS

The effect of “2,3-DHBA + PCS” chemosensitization was also examined in the *Candida* where “no synergistic” FICIs were found. However, there was increased antifungal activity with 2,3-DHBA + PCS (*i.e.*, chemosensitization) in five strains (*i.e.*, all three *C. krusei* strains, *C. albicans* CAN242 and *C. glabrata* CAN252)*,* as reflected in lower MICs of each compound when co-applied (Table 6) than when applied alone. However, FICIs for the remaining four *Candida* strains and FFCIs for all *Candida* were 2.0 (*i.e.*, no compound interactions occurred at all), indicating these fermenting yeast strains were relatively tolerant to any chemosensitization exerted by “2,3-DHBA + PCS.”

**Table 6:** Antifungal chemosensitization of 2,3-DHBA (mM) to PCS (μg/mL) tested against *Candida*: EUCAST-based microdilution bioassays.

Strains	Compounds	MIC	MIC	FICI
		alone	combined	
*C. albicans* 90028	2,3-DHBA	0.2	0.2	2.0
	PCS	>16 ^a^	>16	
*C. albicans* CAN242	2,3-DHBA	0.4	0.2	0.6
	PCS	>16	2	
*C. albicans* CAN276	2,3-DHBA	0.2	0.2	2.0
	PCS	>16	>16	
*C. glabrata* 90030	2,3-DHBA	0.2	0.2	2.0
	PCS	>16	>16	
*C. glabrata* 2001	2,3-DHBA	0.2	0.2	2.0
	PCS	>16	>16	
*C. glabrata* CAN252	2,3-DHBA	0.4	0.2	0.6
	PCS	>16	2	
*C. krusei* 6258	2,3-DHBA	0.4	0.2	0.6
	PCS	>16	2	
*C. krusei* CAN82	2,3-DHBA	0.4	0.2	0.6
	PCS	>16	2	
*C. krusei* CAN75	2,3-DHBA	0.4	0.2	0.6
	PCS	>16	2	
Mean	2,3-DHBA	0.3	0.2	1.1
	PCS	32.0	15.3	
*t*-test ^b^	2,3-DHBA	-	*p *< 0.01	-
	PCS	-	*p *< 0.01	-
*C. albicans* 90028	2,3-DHBA	6.4	6.4	2.0
	PCS	>16	>16	
*C. albicans* CAN242	2,3-DHBA	3.2	3.2	2.0
	PCS	>16	>16	
*C. albicans* CAN276	2,3-DHBA	>6.4	>6.4	2.0
	PCS	>16	>16	
*C. glabrata* 90030	2,3-DHBA	1.6	1.6	2.0
	PCS	>16	>16	
*C. glabrata* 2001	2,3-DHBA	3.2	3.2	2.0
	PCS	>16	>16	
*C. glabrata* CAN252	2,3-DHBA	1.6	1.6	2.0
	PCS	>16	>16	
*C. krusei* 6258	2,3-DHBA	6.4	6.4	2.0
	PCS	>16	>16	
*C. krusei* CAN82	2,3-DHBA	>6.4	>6.4	2.0
	PCS	>16	>16	
*C. krusei* CAN75	2,3-DHBA	>6.4	>6.4	2.0
	PCS	>16	>16	
Mean	2,3-DHBA	6.8	6.8	2.0
	PCS	32.0	32.0	
*t*-test	2,3-DHBA	-	*p* = 1.0	-
	PCS	-	N/D ^c^	-

^a^ PCS was tested up to 16 μg/mL. For calculation purposes, 32 μg/mL (doubling of 16 μg/mL) was used. ^b^ Student’s *t*-test for paired data, mean MIC or MFC of each compound (combined, *i.e*., chemosensitization) *vs*. mean MIC or MFC of each compound (alone, *i.e*., no chemosensitization), was determined in nine strains (Calculation was based on [36]). ^c^ N/D, not determined (all > 16 μg/mL).

#### 2.2.4. Chemosensitization in *Cryptococcus*: OG + PCS

Next, chemosensitization efficacy of “OG + PCS” in *Cryptococcus* was evaluated. For MICs, synergistic FICIs were achieved for most of the *Cryptococcus* strains (Table 7). Similar to the results for “2,3-DHBA + PCS”, the only exception for achieving synergism was *C. neoformans* 4567, which was determined to be an “indifferent” interaction. However, increased antifungal activity of OG and PCS (*i.e.*, chemosensitization; FICI = 0.6) could be achieved in *C. neoformans* 4567*,* resulting in lowered MICs of OG or PCS when co-applied.

For MFCs, synergistic FFCIs (at the level of ≥ 99.9% fungal death) between OG and PCS occurred in all *Cryptococcus* strains (Table 7), reflecting the most potent antifungal activity of OG, as determined in Table 4. Most notable is that the concentration of OG needed to achieve synergism with PCS was much lower than that for 2,3-DHBA, *i.e.*, chemosensitizing potency (higher to lower, as indicated by lower concentrations required) = OG (0.01–0.02 mM) > 2,3-DHBA (0.2–1.6 mM; See also Table 5).

**Table 7:** Antifungal chemosensitization of OG (mM) to PCS (μg/mL) tested against *Cryptococcus* strains: EUCAST-based microdilution bioassays. ^a^

Strains	Compounds	MIC	MIC	FICI	
		alone	combined		
*C. neoformans* 90112	OG	0.04	0.01	**0.4**
	PCS	4	0.5	
*C. neoformans* 208821	OG	0.04	0.01	**0.3**
	PCS	>16 ^b^	0.5	
*C. neoformans* 4564	OG	0.04	0.01	**0.4**
	PCS	>16	4	
*C. neoformans* 4565	OG	0.04	0.01	**0.3 **
	PCS	>16	0.5	
*C. neoformans* 4566	OG	0.04	0.01	**0.3**
	PCS	>16	1	
*C. neoformans* 4567	OG	0.04	0.02	0.6
	PCS	4	0.25	
*C. neoformans* CN24	OG	0.04	0.01	**0.5**
	PCS	16	4	
*C. gatti* 4560	OG	0.02	0.01	**0.5**
	PCS	8	0.25	
*C. gatti* 4561	OG	0.04	0.01	**0.4**
	PCS	4	0.5	
Mean	OG	0.04	0.01	**0.3**
	PCS	18.2	1.3	
*t*-test ^c^	OG	-	*p *< 0.005	-
	PCS	-	*p *< 0.005	-
*C. neoformans *90112	OG	0.04	0.01	**0.4**
	PCS	>16	4	
*C. neoformans *208821	OG	0.04	0.01	**0.3**
	PCS	>16	2	
*C. neoformans *4564	OG	0.04	0.02	**0.5**
	PCS	>16	0.25	
*C. neoformans *4565	OG	0.04	0.02	**0.5**
	PCS	>16	0.25	
*C. neoformans *4566	OG	0.04	0.01	**0.5**
	PCS	>16	8	
*C. neoformans *4567	OG	0.04	0.02	**0.5**
	PCS	>16	0.25	
*C. neoformans* CN24	OG	0.08	0.02	**0.4**
	PCS	>16	4	
*C. gatti* 4560	OG	0.04	0.01	**0.3**
	PCS	>16	0.5	
*C. gatti* 4561	OG	0.04	0.01	**0.4**
	PCS	>16	4	
Mean	OG	0.04	0.01	**0.3**
	PCS	32.0	2.6	
*t*-test	OG	-	*p *< 0.005	-
	PCS	-	*p *< 0.005	-

^a^ Synergistic FICIs and FFCIs are indicated in bold; ^b^ PCS was tested up to 16 μg/mL. For calculation purposes, 32 μg/mL (doubling of 16 μg/mL) was used; ^c^ Student’s *t*-test for paired data, mean MIC or MFC of each compound (combined, *i.e*., chemosensitization) *vs*. mean MIC or MFC of each compound (alone, *i.e*., no chemosensitization), was determined in nine strains (Calculation was based on [36]).

#### 2.2.5. Chemosensitization in *Candida*: OG + PCS

The chemosensitization effect of “OG + PCS” was further examined in the *Candida* strains. For MICs, “synergistic” FICIs were found in four strains, *i.e*., all three *C. krusei* strains and *C. glabrata* CAN252 (Table 8). This synergism was not detected with “2,3-DHBA + PCS”, further reflecting the higher antifungal activity of OG than 2,3-DHBA (See also Table 6). Despite the “indifferent” interaction, increased antifungal activity of OG and PCS (*i.e.*, chemosensitization; FICI = 0.6) occurred in *C. albicans* CAN242*,* as determined in lowered MICs of OG or PCS when combined (Table 8).

Of note, the trends of compound interactions of OG + PCS for MICs in Table 8 were congruent with “2,3-DHBA + PCS” chemosensitization (Table 6). For both “2,3-DHBA + PCS” and “OG + PCS”, incremental increase of growth inhibition occurred in five common strains, *i.e*., all three *C. krusei* strains, *C. albicans* CAN242 and *C. glabrata* CAN252 (Table 6, Table 8). This indicated that strain specificity to chemosensitization also exists. This is reflected in the level of differential vulnerability of each strain to chemosensitization (See also FICIs of *C. neoformans* 4567 in Table 5, Table 7, showing lower sensitivity of this strain to both OG- and 2,3-DHBA-mediated chemosensitization compared to other *Cryptococcus* strains).

For MFCs, synergistic FFCIs (at the level of ≥99.9% fungal death) between OG and PCS were achieved in *C. krusei* CAN82 and *C. glabrata* CAN252. While the FFCI of *C. krusei* 6258 was scored as “indifferent”, there was increased antifungal activity of OG and PCS (*i.e.*, chemosensitization; FFCI = 0.6) with this strain. FFCIs for the remaining strains were “indifferent”, and as observed in “2,3-DHBA + PCS” assays, *Candida* strains were more tolerant to “OG + PCS” chemosensitization compared to *Cryptococcus* strains.

**Table 8:** Antifungal chemosensitization of OG (mM) to PCS (μg/mL) tested against *Candida* strains: EUCAST-based microdilution bioassays. ^a^

Strains	Compounds	MIC	MIC	FICI
		alone	combined	
*C. albicans* 90028	OG	0.08	0.08	2.0
	PCS	>16 ^b^	>16	
*C. albicans* CAN242	OG	0.08	0.04	0.6
	PCS	>16	2	
*C. albicans* CAN276	OG	0.08	0.08	2.0
	PCS	>16	>16	
*C. glabrata* 90030	OG	0.04	0.04	2.0
	PCS	>16	>16	
*C. glabrata* 2001	OG	0.04	0.04	2.0
	PCS	>16	>16	
*C. glabrata* CAN252	OG	0.08	0.02	**0.3**
	PCS	>16	2	
*C. krusei* 6258	OG	0.08	0.04	**0.5**
	PCS	>16	0.25	
*C. krusei* CAN82	OG	0.08	0.04	**0.5**
	PCS	>16	0.25	
*C. krusei* CAN75	OG	0.08	0.04	**0.5**
	PCS	>16	0.5	
Mean	OG	0.07	0.05	1.2
	PCS	32.0	14.8	
*t*-test ^c^	OG	-	*p *< 0.05	-
	PCS	-	*p *< 0.01	-
*C. albicans* 90028	OG	0.08	0.08	2.0
	PCS	>16	>16	
*C. albicans* CAN242	OG	0.08	0.08	2.0
	PCS	>16	>16	
*C. albicans* CAN276	OG	0.08	0.08	2.0
	PCS	>16	>16	
*C. glabrata* 90030	OG	0.08	0.08	2.0
	PCS	>16	>16	
*C. glabrata* 2001	OG	0.08	0.08	2.0
	PCS	>16	>16	
*C. glabrata* CAN252	OG	0.08	0.04	**0.5**
	PCS	>16	1	
*C. krusei* 6258	OG	0.08	0.04	0.6
	PCS	>16	4	
*C. krusei* CAN82	OG	0.16	0.08	**0.5**
	PCS	>16	0.25	
*C. krusei* CAN75	OG	0.16	0.16	2.0
	PCS	>16	>16	
Mean	OG	0.1	0.08	1.5
	PCS	32.0	21.9	
*t*-test	OG	-	*p *< 0.5	-
	PCS	-	*p *< 0.1	-

^a^ Synergistic FICIs and FFCI are indicated in bold. ^b^ PCS was tested up to 16 μg/mL. For calculation purposes, 32 μg/mL (doubling of 16 μg/mL) was used. ^c^ Student’s *t*-test for paired data, mean MIC or MFC of each compound (combined, *i.e*., chemosensitization) *vs*. mean MIC or MFC of each compound (alone, *i.e*., no chemosensitization), was determined in nine strains (Calculation was based on [36]).

The results of all chemosensitization tests (*i.e*., PCS + 2,3-DHBA or OG in both *Cryptococcus* and *Candida* strains) are summarized in Table 9. Exemplary MFC test results, based on chemosensitization (PCS + OG or 2,3-DHBA) performed in *Cryptococcus* or *Candida* strains, are provided in Figure 3.

**Table 9:** SUMMARY: Antifungal chemosensitization of OG or 2,3-DHBA (mM) to PCS (μg/mL) tested against *Cryptococcus* or *Candida* strains determined by EUCAST-based microdilution bioassays. Data shown are mean values derived from Table 5, Table 6, Table 7, Table 8. ^a^

	Compounds	MIC	MIC	FICI	MFC	MFC	FFCI
		alone	combined		alone	combined	
Cryptococcus	OG	0.04	0.01	**0.3**	0.04	0.01	**0.3**
	PCS	18.2	1.3		32.0	2.6	
Candida	OG	0.07	0.05	1.2	0.1	0.08	1.5
	PCS	32.0	14.8		32.0	21.9	
Cryptococcus	2,3-DHBA	0.2	0.05	**0.4**	1.5	0.8	0.9
	PCS	18.2	1.9		32.0	11.6	
Candida	2,3-DHBA	0.3	0.2	1.1	6.8	6.8	2.0
	PCS	32.0	15.3		32.0	32.0	

^a^ Synergistic FICIs and FFCI are indicated in bold.

#### 2.2.6. Differential Responses of *S. cerevisiae* Antioxidant Mutants to OG or 2,3-DHBA

Identification of fungal target(s) of OG or 2,3-DHBA within the antioxidant system was attempted using gene deletion mutants of the model yeast, *S. cerevisiae*. Agar plate-based yeast dilution bioassays on SG with OG or 2,3-DHBA (See Experimental section for test concentrations), in duplicate, included the wild type (WT) and five antioxidant mutant strains, as follows: (1) *yap1*Δ (Yap1p is the transcription factor regulating expression of four downstream genes within the oxidative stress response pathway, *i.e.*, *GLR1* [glutathione reductase], *YCF1* [a glutathione *S*-conjugate pump], *GSH1* [γ-glutamylcysteine synthetase, which catalyzes the first step in glutathione biosynthesis] and *TRX2* [thioredoxin] [37,38]), (2) *sod2*Δ (mitochondrial superoxide dismutase, Mn-SOD), (3) *trr1*Δ (cytoplasmic thioredoxin reductase), (4) *trr2*Δ (mitochondrial thioredoxin reductase), and (5) *tsa1*Δ (thioredoxin peroxidase) (See *Saccharomyces* Genome Database [30]).

**Figure 4:**
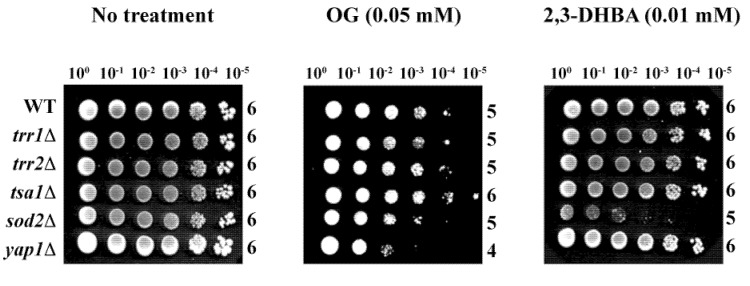
Agar plate-based yeast dilution bioassay identifying sensitive mutants of *S. cerevisiae* to phenolic chemosensitizers (10^0^ to 10^−5^: yeast dilution rates). Results showed the sensitive responses of *yap1*Δ to OG and *sod2*Δ to 2,3-DHBA, respectively.

Of the five deletion mutants, *yap1*Δ was hypersensitive to OG (log_10_ = 4), while *sod2*Δ was hypersensitive to 2,3-DHBA (log_10_ = 5) (Figure 4) (see also [15]). These results indicate OG or 2,3-DHBA affect different cellular components in fungi, where Mn-SOD plays a relatively greater role in fungal tolerance to 2,3-DHBA, while glutathione homeostasis, *etc*., protects cells from OG-induced toxicity, compared to the other genes represented. Further studies, such as microarray-based chemogenomic analysis, inclusion of more gene deletion mutants, *etc*., are warranted to determine the precise mechanism of action of OG or 2,3-DHBA during chemosensitization.

## 3. Experimental

### 3.1. Yeast Strains

Yeast strains (*Cryptococcus*-*C. neoformans*, *C. gatti*; *Candida*- *C. albicans*, *C. glabrata*, *C. krusei*; *Saccharomyces cerevisiae*; See Table 2) were cultured on synthetic glucose (SG; yeast nitrogen base without amino acids 0.67%, glucose 2% with appropriate supplements: uracil 0.02 mg/mL, amino acids 0.03 mg/mL) or Yeast Peptone Dextrose (YPD; Bacto yeast extract 1%, Bacto peptone 2%, glucose 2%) medium at 35 °C for yeast pathogens (*Candida*, *Cryptococcus*) or 30 °C for *S. cerevisiae*, respectively.

### 3.2. Chemicals

The chemosensitizing agents (octyl gallate [OG], propyl gallate [PG], 2,3-dihydroxybenzaldehyde [2,3-DHBA], acetylsalicylic acid [AcSA], proguanil) and inhibitors of mitochondrial respiratory chain (MRC) (rotenone, carboxin, thenoyltrifluoroacetone [TTFA], 3-nitropropionic acid [3-NPA], benzhydroxamic acid [BHAM], salicylhydroxamic acid [SHAM], antimycin A [AntA], kresoxim methyl [Kre-Me], pyraclostrobin [PCS], azoxystrobin [AZS], potassium cyanide [KCN], sodium azide [Na-azide], atovaquone [ATQ]) were procured from Sigma Co. (St. Louis, MO, USA). Each compound was dissolved in dimethyl sulfoxide (DMSO; absolute DMSO amount: < 1% in media) before incorporation into culture media. In all tests, control plates (*i.e*., “No treatment”) contained DMSO at levels equivalent to that of cohorts receiving antifungal agents, within the same set of experiments.

### 3.3. Antifungal Bioassay: Microtiter Plate (Microdilution) Bioassay

Chemosensitizing activity of OG (0.005, 0.01, 0.02, 0.04, 0.08, 0.16, 0.32, 0.64 mM) or 2,3-DHBA (0.05, 0.1, 0.2, 0.4, 0.8, 1.6, 3.2, 6.4 mM) to PCS (0.25, 0.5, 1, 2, 4, 8, 16 µg/mL) was determined by using checkerboard bioassays in microtiter plates (with RPMI 1640 medium; Sigma Co.). To determine changes in MICs of antifungal agents (*i.e*., PCS and chemosensitizers) in microtiter wells, checkerboard bioassays (0.5 × 10^5^ to 2.5 × 10^5^ CFU/mL) were performed using broth microdilution protocols according to methods outlined by the European Committee on Antimicrobial Susceptibility Testing (EUCAST) [32]. Minimum inhibitory concentration (MIC) for chemosensitization was defined as the lowest concentration of agent(s) where no fungal growth was visible at 24 and 48 h. All bioassays were performed in triplicate. Statistical analysis was based on [36]. Microtiter plate (microdilution) bioassay was also performed to determine chemosensitizing activity of proguanil (0.25, 0.5, 1, 2, 4, 8, 16 μg/mL) to ATQ (0.25, 0.5, 1, 2, 4, 8, 16 μg/mL). Minimum fungicidal concentration (MFC), which is the lowest concentration of agents exhibiting ≥ 99.9% fungal death, were determined (after completion of MIC assays) wherein entire volumes of microtiter wells (200 µL) were spread onto individual YPD plates, and cultured for another 48 and 72 h. Compound interactions, Fractional Inhibitory Concentration Indices (FICI) and Fractional Fungicidal Concentration Indices (FFCI) were calculated, as follows: FICI or FFCI = (MIC or MFC of compound A in combination with compound B/MIC or MFC of compound A, alone) + (MIC or MFC of compound B in combination with compound A/MIC or MFC of compound B, alone). Interactions were defined as: “synergistic” (FICI or FFCI ≤ 0.5) or “indifferent” (FICI or FFCI > 0.5–4) [34].

### 3.4. Antifungal Bioassay: Agar Plate (Yeast Dilution) Bioassay

Petri plate-based yeast dilution bioassays were performed on the wild type and antioxidant mutants (*trr1*Δ, *trr2*Δ, *tsa1*Δ, sod2Δ, *yap1*Δ) to assess the effects of OG (0.005, 0.01, 0.015, 0.02, 0.025, 0.03, 0.035, 0.04, 0.045, 0.05, 0.1 mM) and 2,3-DHBA (0.005, 0.01, 0.015, 0.02, 0.025, 0.03, 0.035, 0.04, 0.045, 0.05, 0.1, 0.2, 0.3 mM) on the antioxidant system. Similar yeast dilution bioassays were also performed on strains of *Cryptococcus* or *Candida* to assess antifungal capacity/effects of OG (0.1, 0.2, 0.3, 0.4, 0.5, 0.6, 0.7, 0.8, 0.9, 1.0 mM), 2,3-DHBA (0.0125, 0.025, 0.05, 0.1, 0.15, 0.2, 0.25, 0.3, 0.4, 0.5, 1.0, 2.0, 3.0 mM), PG (1, 2, 3, 4, 5, 6, 7, 8, 9, 10 mM), AcSA (1, 2, 3, 4, 5, 6, 7, 8, 9, 10 mM) or MRC inhibitors (100 μM).

These assays were performed in duplicate on SG agar following previously described protocols [15] as follows: 1 × 10^6^ cells of the wild type or gene deletion mutants of *S. cerevisiae*, *Cryptococcus* or *Candida*, cultured on YPD, were serially diluted 10-fold in SG liquid medium (supplemented with amino acids and uracil, if required) five times, which yields cell dilution cohorts of 10^6^, 10^5^, 10^4^, 10^3^, 100 and 10 cells. Cells from each dilution of respective yeast strains were spotted adjacently on SG agar medium incorporated with antifungal compounds to be tested and incubated at 30 °C or 35 °C for *S. cerevisiae* or *Cryptococcus*/*Candida*, respectively. Results were monitored based on a designated log_10_ score of the highest dilution where a colony became visible after 3–5 days of incubation, as follows: Score ‘0’—no colonies were visible from any of the dilutions, Score ‘6’—colonies were visible from all dilutions, Score ‘1’—only a colony from the undiluted cells (*i.e.*, 10^6^ cells), ‘2’ only colonies from the undiluted and 10^5^ cells were visible, *etc*. Therefore, each unit of numerical difference (e.g., 10^2^ *vs*. 10^3^) was equivalent to a 10-fold difference in the sensitivity of the yeast strain to the treatment.

### 3.5. Statistical Analysis

Statistical analyses, e.g., chemosensitization *vs*. no chemosensitization, were performed based on [36].

## 4. Conclusions

In summary, OG- or 2,3-DHBA-based chemosensitization can enhance antifungal activity of PCS in *Cryptococcus* and *Candida*. Our results showed that: (1) All *Cryptococcus* strains (non-fermentors) were sensitive to PCS + OG or 2,3-DHBA; (2) Only selected *Candida* strains (three *C. krusei* strains, *C. albicans* CAN242, *C. glabrata* CAN252) (fermentors) were sensitive to PCS + OG or 2,3-DHBA; (3) OG was a more potent chemosensitizer than 2,3-DHBA to PCS, where the concentration of OG required to achieve “synergism” was much lower (≥20 times lower) than 2,3-DHBA in either *Cryptococcus* or *Candida* strains; (4) “chemosensitization - strain specificity” exists, which reflects differential vulnerability of tested strains to the chemosensitization; (5) OG or 2,3-DHBA disrupt different cellular components in fungi, where Mn-SOD plays a role in fungal tolerance to 2,3-DHBA, while glutathione homeostasis, *etc*., are responsible for protecting cells from OG-triggered toxicity.

The MRC is recently recognized as a new target for development of clinical antimycotics [24,39]. For example, co-application of AntA (MRC-inhibitory) and BHAM (AOX-inhibitory) significantly increased the activity of triazole drugs, potentiating the antifungal activity of the drugs as fungicidal in *R. oryzae* (causative agent of mucormycosis) [24]. Also, inhibition of MRC of *C. parapsilosis* (causing neonatal and device-related infections; See [39] and references therein) enhances susceptibility of this fungal pathogen to caspofungin, a cell wall-targeting drug [39]. Thus, use of a chemosensitizer, as described in this study, would lower the effective dose of an MRC-inhibitory drug, thus lowering potential side effects of these drugs and others that might be co-applied (*i.e.*, azoles, caspofungin, *etc*.). This lower dosage would render treatment less expensive and safer, thus making their use more acceptable.

In conclusion, OG and/or 2,3-DHBA show potential to serve as antifungal chemosensitizers that in combination with PCS greatly enhance antifungal activity. This capacity was shown to be most effective against *Cryptococcus*, etiologic agents for the leading cause of death among those suffering from immunocompromised disorders. Chemosensitizers, especially those proven to be safe compounds, such as natural phenolic agents or their structural derivatives, could serve as potential “leads” against yeast pathogens for more effective treatment of mycoses using MRC inhibitory drugs. Determination of precise mechanisms of action of chemosensitization as well as identification of effective MRC-inhibitory drugs which selectively interfere with fungal mitochondrial function, and not human (mammalian), must be ensured through future study.
